# Transcriptome profiling of human oocytes experiencing recurrent total fertilization failure

**DOI:** 10.1038/s41598-018-36275-6

**Published:** 2018-12-17

**Authors:** Lun Suo, Yu xiao Zhou, Li ling Jia, Hai bo Wu, Jin Zheng, Qi feng Lyu, Li hua Sun, Han Sun, Yan ping Kuang

**Affiliations:** 1 Department of Assisted Reproduction, Shanghai Ninth People’s Hospital, Shanghai Jiao Tong University School of Medicine, Shanghai, China; 2 Center for Comparative Biomedicine, MOE Key Laboratory of Systems Biomedicine, Institute of Systems Biomedicine, SCSB, Shanghai Jiao Tong University (SJTU), Shanghai, 200240 China; 3 Department of Genetics, School of Medicine, Stanford University, Stanford, CA 94305 USA

## Abstract

There exist some patients who face recurrent total fertilization failure during assisted reproduction treatment, but the pathological mechanism underlying is elusive. Here, by using sc-RNA-seq method, the transcriptome profiles of ten abnormally fertilized zygotes were assessed, including five zygotes from one patient with recurrent Poly-PN zygotes, and five zygotes from a patient with pronuclear fusion failure. Four zygotes with three pronuclear (Tri-PN) were collected from four different patients as controls. After that, we identified 951 and 1697 significantly differentially expressed genes (SDEGs) in Poly-PN and PN arrest zygotes, respectively as compared with the control group. KEGG analyses indicated down regulated genes in the Poly-PN group included oocyte meiosis related genes, such as *PPP2R1B, YWHAZ, MAD2L1, SPDYC, SKP1* and *CDC27*, together with genes associated with RNA processing, such as *SF3B1, LOC645691, MAGOHB, PHF5A, PRPF18, DDX5, THOC1* and *BAT1*. In contrast, down regulated genes in the PN arrest group, included cell cycle genes, such as *E2F4, DBF4, YWHAB, SKP2, CDC23, SMC3, CDC25A, CCND3, BUB1B, MDM2, CCNA2* and *CDC7*, together with homologous recombination related genes, such as *NBN, XRCC3, SHFM1, RAD54B* and *RAD51*. Thus, our work provides a better understanding of transcriptome profiles underlying RTFF, although it based on a limited number of patients.

## Introduction

Despite nearly forty years of scientific and clinical advance in the field of assisted reproduction, there still exist some rarely patients, even though rarely occur, who have to face recurrent total fertilization failure (RTFF) without any visual precautionary indicator^1–3^, even some of them could be rescued by assisted oocyte activation^4^. Therefore, elucidating the internal mechanism of fertilization failure is of great importance for these patients. However, until now, the relevant etiological analysis was often restricted to morphology during IVF, such as immature oocytes^5^, improper meiosis^6^, zygotes with abnormal pronuclei^7^, and di-pronuclear zygote failing mitotic cleavage^8^. Due to small amount of material available, deciphering mechanisms underlying these defects remain technical challenging.

Recent technical advances in single-cell sequencing open a new era for exploring the biological state of a single cell at both the DNA and RNA levels for studying variations in genome^9,10^, transcriptome, and epigenome^11^ separately or in parallel^12^. Originally adopted by Surani’s research team^13^, this approach has been applied successfully in discriminating cell types^14–19^, elucidating regulatory circuits^20^, and investigating tumor heterogeneity^21,22^. In reproductive biology fields, this technique has been used for screening transcriptome of tissues^23^ and germline cells at different stages^24–29^. The single cell sequencing technique has great potential in clinical implication^30–32^, especially in the diagnosis for clarifying the molecular mechanisms of fertilization failure at a single cell resolution. So the aim of this work was to characterize the pathological changes of human zygotes with RTFF at the transcriptional level.

## Results

### Clinical treatment history of the RTFF patients

As clinical treatment history shown (Table 1), one patient experienced two stimulated cycles under different procedures with 4 and 5 Poly-PN fertilized eggs after ICSI treatment, respectively. Another patient had all zygotes with PN arrest, with 18, 7 and 9 matured oocytes retrieved separately in three cycles although three different ovarian stimulation procedures were employed each time. Moreover, There were no significant differences in serum levels of FSH, LH, E_2_ and progesterone at baseline and trigger day in patients with Poly-PN, PN arrest, and the control groups (Supplementary Fig. 1), indicating that the observed defects in the zygotes were more likely associated with oocyte original molecular defects rather than ovarian stimulation protocol.

**Table 1:** Embryonic developmental consequence of the patients with RTFF during assisted reproduction treatment.

Clinical cycles	Patient with recurred Poly-PN zygotes								
			Oocyte	Oocyte				Fertilization	
				MII	MI	GV	Abnormal	0PN	Poly-PN
1^st^	HMG+MPA+EE	ICSI	11	5	3	2	1	1	4
2^nd^	HMG+MPA+CC	ICSI	7	6	0	1	0	1	5
	Patient with recurred PN-arrest zygotes								
			Oocyte					Fertilization	
			Total	MII	MI	GV	Abnormal	2PN	PN arrest
1^st^	Short protocol	IVF/ICSI	19	9/9	0	0	1	9/9	18
2^nd^	HMG + MPA	ICSI	8	7	0	0	1	7	7
3^rd^	HMG + CC	ICSI	10	9	0	1	0	9	9

### Transcriptome profiles in Poly-PN and PN-arrest zygotes

**Figure 1:**
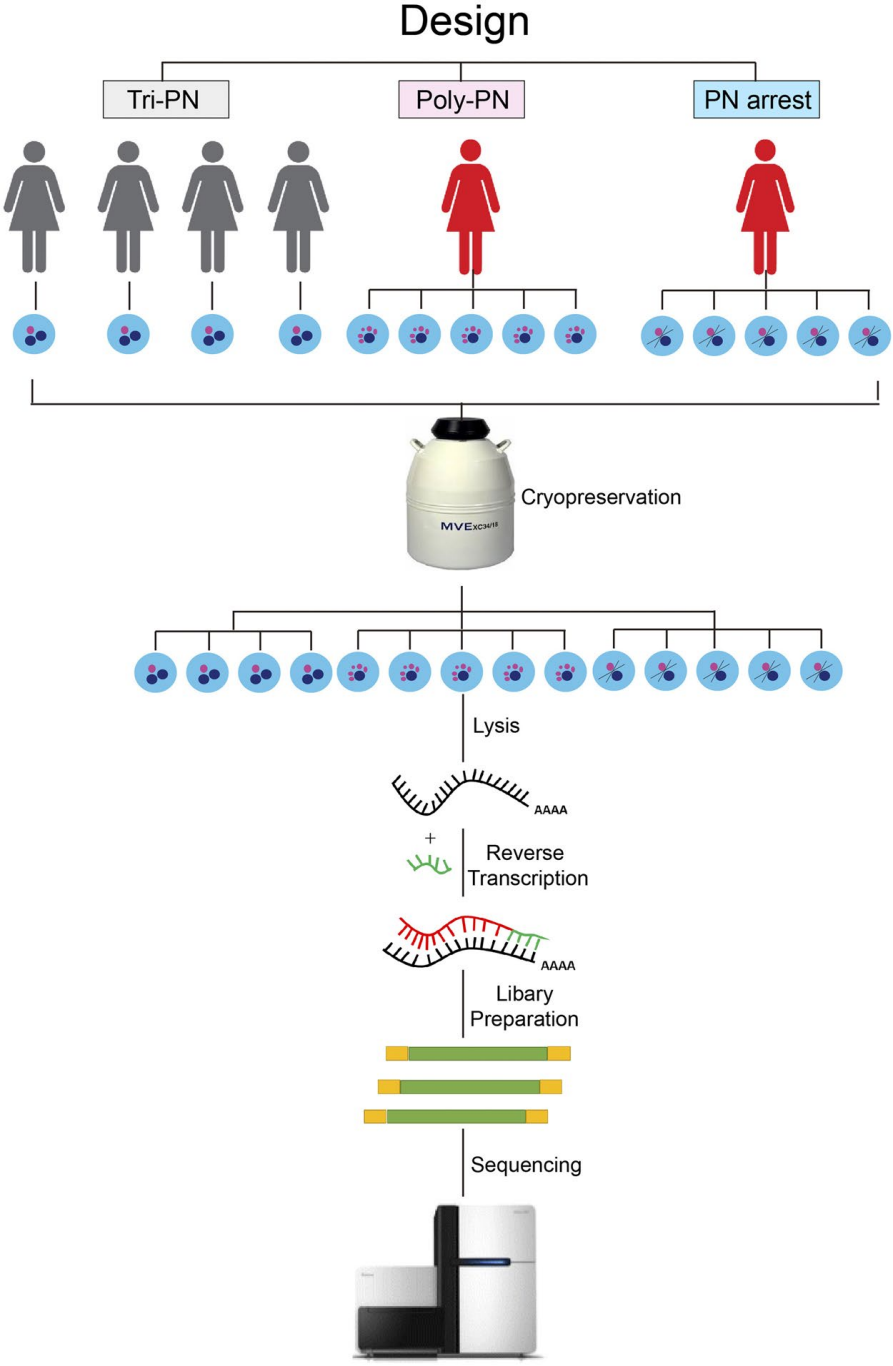
Overview of the scRNA-seq experimental design.

**Figure 2:**
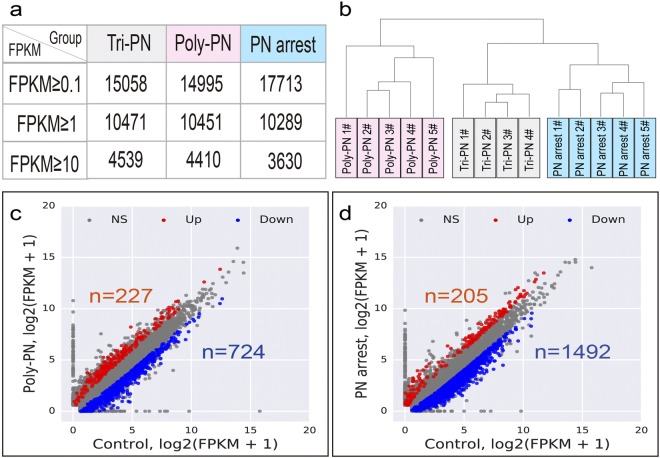
Transcriptome profile of the Poly-PN and PN-arrest zygotes. (**a**) The number of reference transcripts averagely found in each sample in different groups based on clustering of expression patterns for 14 single cell samples from Poly-PN, PN arrest and Control groups; (**c**,**d**) Scatterplot showing the number of genes up regulated (red) and down regulated (blue) in Poly PN (**c**) and PN arrest zygotes (**d**) separately compared with control group.

It is crucial for oocyte to accumulate indispensable mRNAs to ensure its later use for fertilization and subsequent cell division before the zygotic gene activation^33^. As the scarce of the oocytes for RTFF patients, it was difficult to collect enough donated oocytes for our study. Therefore, we investigate the transcriptome profile using the unfertilized oocyte after clinical treatment. The procedure of our work was shown in Fig. 1. After sequencing using Illumina HiSeq 2,500 sequencer, we obtain about 142 million clean reads, of which 116 million clean reads mapped to human genome reference. On average, 15,058, 14,995 and 17,713 genes (FPKM ≥ 0.1), 10,471, 10,451 and 10,289 genes (FPKM ≥ 1) or 4,539, 4,410 and 3,630 genes (FPKM ≥ 10) were acquired in Tri-PN, Poly-PN and PN arrest groups, respectively (Fig. 2a). These results were consistent with the data from a previous study^24^, implying that our technology has the similar sensitivity and coverage.

To compare the global transcriptome profiles of unfertilized eggs or zygotes among different groups, we analyzed data by hierarchical clustering, and the results indicated that 14 zygotes from 3 groups were clustered into corresponding groups and separated from each other (Fig. 2b). Four Tri-PN zygotes from different patients have the similar transcriptome profile in spite of the heterogeneity in patient source. Interestingly, we also found that five PN arrest zygotes clustered closer with four Tri-PN zygotes, but away from the five Poly-PN zygotes. This finding indicated that the underlying mechanisms was quite different between the Poly-PN and PN arrest group.

To clarify underlying mechanisms, we analyzed major differences of expression profile among different groups. To rule out technical errors causing artifacts of gene expression, all reference genes with average FPKM > 0.5 in any of three groups were used for subsequent analysis. According to the criteria of fold change > 2 or < 0.5 and *P* < 0.001, 951 (227 up regulated and 724 down regulated) and 1,697 genes (205 up regulated and 1,492 down regulated) were found to be significantly differentially expressed genes (SDEGs) in Poly-PN and PN arrest zygotes, respectively, as compared with the control group (Fig. 2c,d and Supplementary Tables 1 and 2).

### The SDEGs are involved in different biological processes between Poly-PN and PN arrest zygotes

In order to clarify the biological function of these differential expressed genes, the KEGG analyses were applied to SDEGs of Poly-PN and PN arrest zygotes, separately. As the results shown (Table 2), for the up regulated genes, there was a enrichment of genes whose products are related to RNA processing and translation, such as RNA splicing (*P*-value = 1.90 × 10^−2^ for Poly-PN) or Ribosome biogenesis (*P*-value = 5.80 × 10^−9^ for Poly-PN and 1.10 × 10^−84^ for PN arrest), together with energy consuming related items, such as Huntington’s disease (*P*-value = 6.40 × 10^−3^ for Poly-PN), Parkinson’s disease (*P*-value = 1.20 × 10^−3^ for PN arrest) and Oxidative phosphorylation (*P*-value = 5.20 × 10^−5^ for PN arrest). Genes involved in Wnt signaling pathway (*P*-value = 1.00 × 10^−2^ for Poly-PN), Notch signaling pathway (*P*-value = 2.00 × 10^−2^ for Poly-PN) and some other signaling pathways in cancer were also enriched (Table 2 and Supplementary Tables 3 and 4). For down regulated genes, the Poly-PN specific down regulated genes were mainly enriched in Oocyte meiosis (*P*-value = 3.60 × 10^−2^), Spliceosome (*P*-value = 4.00 × 10^−3^), Pyrimidine metabolism (*P*-value = 1.60 × 10^−2^), Citrate cycle (*P-*value = 3.80 × 10^−3^) (Table 3 and Supplementary Table 5). Whereas, the PN arrest specific down regulated genes mainly belonged to Cell cycle (*P*-value = 7.60 × 10^−3^), Homologous recombination (*P*-value = 2.40 × 10^−2^) and Amino sugar or nucleotide sugar metabolism (*P*-value = 4.60 × 10^−2^) (Table 3 and Supplementary Table 6). Furthermore, The SDEGs down regulated overlapped in both of this two groups were mainly related to Basal transcription factors (*P*-value = 3.10 × 10^−2^ for Poly-PN and 4.20 × 10^−3^ for PN arrest), Ubiquitin mediated proteolysis (*P*-value = 4.70 × 10^−2^ for Poly-PN and 7.70 × 10^−3^ for PN arrest) and Glycan biosynthesis (*P*-value = 2.00 × 10^−2^ for Poly-PN and 3.10 × 10^−2^ for PN arrest).

**Table 2:** KEGG Signaling pathways enrichment of SDEGs up regulated in Poly-PN and PN arrest groups separately compared with controls.

	Items	Count	%	*P*-Value	Genes
SDEGs (Poly-PN/Control)	Ribosome	12	5.5	5.80E-09	RPSA, RPL23, RPL9, RPL35, RPL3, RPS9, RPL7A, RPS11, RPS20, RPS4X, RPS8, RPS24
	Huntington’s disease	8	3.6	6.40E-03	POLR2F, TAF4, EP300, NDUFB8, NDUFA6, CREBBP, UQCRQ, ATP5J
	Wnt signaling pathway	7	3.2	1.00E-02	PRKCA, CTBP1, TCF7, EP300, CREBBP, FRAT1, FRAT2
	Pathway in cancer	10	4.5	1.90E-02	PRKCA, CTBP1, TCF7, EP300, RALBP1, CREBBP, FOXO1, LAMC1, IKBKB, LAMB1
	Spliceosome	6	2.7	1.90E-02	CHERP, PCBP1, LSM7, SNRPD2, SNRPG, SF3B2
	Notch signaling pathway	4	1.8	2.00E-02	CTBP1, EP300, PSEN2, CREBBP
	Prostate cancer	5	2.3	2.40E-02	TCF7, EP300, CREBBP, FOXO1, IKBKB
SDEGs (PN arrest/Control)	Ribosome	56	28.1	1.10E-84	RPL36A, RPL19, RPL13, RPLP2, RPS2, RPS3, RPS3A, RPLP0, RPLP1, RPL10, RPL12, RPS27A, RPS4X, RPS18, RPS19, RPL41, RPS16, RPS17, RPS15, RPS12, RPS13, RPS11, UBA52, RPL35, RPS15A, RPL36, RPL37, RPL38, RPS25, RPL30, RPS27, RPS28, RPL32, RPS29, RPL31, RPL8, RPL3, RPL10A, RPL7A, RPL4, RPS20, RPS21, RPS23, RPS24, RPL26, RPS9, RPL27, RPL23A, RPL24, RPS5, RPL28, RPL29, RPL23, RPL13A, RPL37A
	Oxidative phosphorylation	11	5.5	5.20E-05	ATP5D, NDUFA2, NDUFB10, NDUFB8, NDUFB9, NDUFA6, COX6A1, ATP5L, COX6C, NDUFA11, NDUFB2
	Parkinson’s disease	9	4.5	1.20E-03	ATP5D, NDUFA2, NDUFB10, NDUFB8, NDUFB9, NDUFA6, COX6A1, COX6C, NDUFB2
	Alzheimer’s disease	9	4.5	5.50E-03	ATP5D, NDUFA2, NDUFB10, NDUFB8, NDUFB9, NDUFA6, COX6A1, COX6C, NDUFB2
	Huntington’s disease	9	4.5	9.90E-03	ATP5D, NDUFA2, NDUFB10, NDUFB8, NDUFB9, NDUFA6, COX6A1, COX6C, NDUFB2

**Table 3:** KEGG Signaling pathways enrichment of SDEGs down regulated in Poly-PN and PN arrest groups separately compared with controls.

	Items	Count	%	*P*-Value	Genes
SDEGs (Poly-PN/Control)	Citrate cycle (TCA cycle)	6	0.9	0.0038	LOC642502, DLST, SUCLG1, DLD, PDHA2, FH
	Spliceosome	12	1.7	0.004	SFRS7, SF3B1, LOC645691, MAGOHB, PHF5A, HNRNPC, PRPF18, DDX5, SF3B4, PRPF38A, THOC1, BAT1
	Pyrimidine metabolism	9	1.3	0.016	POLR2H, UMPS, POLR2E, RRM2, PNPT1, DCK, ZNRD1, POLR2D, DUT
	N-Glycan biosynthesis	6	0.9	0.02	TUSC3, ALG3, DPM1, ALG6, MGAT5, ALG13
	Basal transcription factors	5	0.7	0.031	TAF9B, GTF2A1L, GTF2B, TBPL1, GTF2H1
	Oocyte meiosis	9	1.3	0.036	PPP2R1B, YWHAZ, MAD2L1, CCNB2, PPP2CA, FBXO5, SKP1, CDC27, SPDYC
	Ubiquitin mediated proteolysis	10	1.4	0.047	UBE2N, UBE2E3, UBE2D3, UBA3, UBE2W, PIAS1, SKP1, UBOX 5, TRAF6, CDC27
SDEGs (PN arrest/Control)	Basal transcription factors	8	0.6	4.20E-03	TAF11, GTF2E1, GTF2I, TAF4B, GTF2F2, TAF9B, GTF2B, GTF2H1
	Cell cycle	16	1.1	7.60E-03	CDC7, E2F4, DBF4, YWHAB, SKP2, CDC23, ANAPC10, SMC3, CDC25A, ORC2L, CCND3, ORC4L, BUB1B, ORC5L, MDM2, CCNA2
	Ubiquitin mediated proteolysis	17	1.2	7.70E-03	UBE2G1, SKP2, CDC23, UBE2F, ANAPC10, UBE2H, UBE2C, BIRC2, BRCA1, UBE2E3, TRIM32, UBA3, UBE2W, MDM2, PIAS2, RCHY1, TRAF6
	Proteasome	8	0.6	2.10E-02	PSMA1, PSMC6, PSMD12, PSMA4, PSMC1, SHFM1, PSMA7, PSMD7
	Homologous recombination	6	0.4	2.40E-02	NBN, XRCC3, RAD51L1, SHFM1, RAD54B, RAD51
	O-Glycan biosynthesis	6	0.4	3.10E-02	GALNT3, GALNT1, C1GALT1C1, GALNT11, GCNT1, C1GALT1
	Amino sugar and nucleotide sugar metabolism	7	0.5	4.60E-02	GNPDA1, GNPDA2, HEXB, UGDH, NAGK, FPGT, UGP2

**Figure 3:**
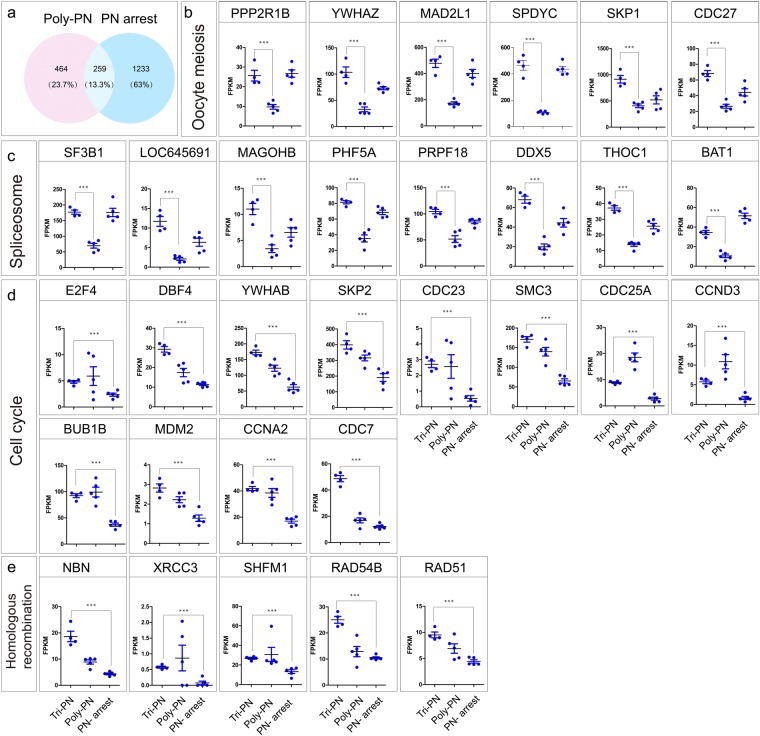
Relative expression levels of SDEGs specifically expressed in Poly-PN and or PN arrest zygotes. (**a**) The Venn diagram of down regulated SDEGs in Poly-PN and PN arrest groups separately compared with those in control group; (**b**–**e**) Scatterplot showing the relative expression levels of the particularly interesting SDEGs specifically down regulated in Poly-PN or PN arrest zygotes. *P* < 0.001 was indicated significantly different.

Among the total 1,956 down regulated genes in different annotations, there were about 464 (23.7%) genes specifically down regulated in Poly PN, 1,233 (63.0%) genes specifically down regulated in PN arrest and only 259 (13.3%) genes down regulated overlap in both of this two groups (Fig. 3a). The Poly-PN specific down regulated genes included oocyte meiosis related genes such as Protein Phosphatase (*PPP2R1B*), *YWHAZ, MAD2L1, SPDYC, SKP1* and *CDC27* (Fig. 3b). Certain genes associated with RNA processing, such as those encoding splicing factor genes *SF3B1, LOC645691, MAGOHB, PHF5A, PRPF18, DDX5, THOC1* and *BAT1* were also down regulated (Fig. 3c), perhaps contributing to the fertilization failure in Poly-PN group. In contrast, the PN arrest specific down regulated genes, such as *E2F4*, *DBF4, YWHAB, SKP2, CDC23, SMC3, CDC25A, CCND3, BUB1B, MDM2, CCNA2, CDC7* were involved in Cell cycle (Fig. 3d) and *NBN, XRCC3, SHFM1, RAD54B, RAD51* were Homologous recombination related genes (Fig. 3e). These results implied that the Poly-PN might have defects during oocyte meiosis, whereas defects of PN arrest zygotes were involved in Cell cycle and Homologous recombination.

### Functional validation of the selected genes by gene knock down

**Figure 4:**
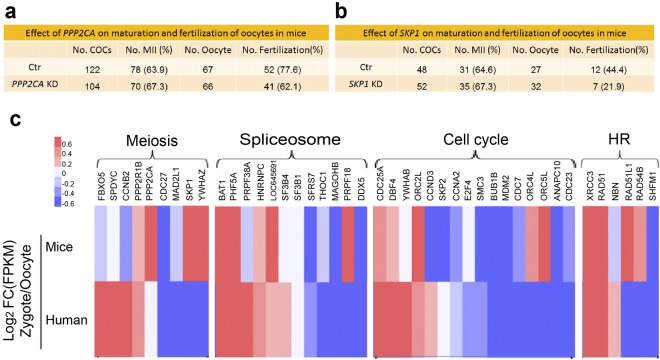
(**a**,**b**) Validation of two meiosis ralated genes in mice oocytes using gene knock down experiment. (**c**) Comparative expression of these identified genes during fertilization in human (bottom layer) and mice (up layer), respectively. HR, Homologous recombination.

We randomly chose two of these meiosis related genes (PPP2CA and SKP1) and validated their function in mice oocyte, the results indicated that both PPP2CA and SKP1 knock down did not show any difference with the corresponding control group in either oocyte maturation or fertilization (Fig. 4a,b). In order to clarify the mechanism underlying, we analyzed available published single cell RNA-Seq data sets corresponding to fertilization process of human and mice, focusing on the 43 selected genes, which enriched in Meiosis, Spliceosome, Cell cycle and Homologous recombination items separately. As the results shown (Fig. 4c), human and mice have very different expression patterns of the selected genes during their fertilization process. Thus, more clinical cases, but not the mice, might be an ideal model for validation of the function of these selected genes in future.

## Discussion

Some patients have oocytes incapable of completing the whole process of fertilization, including defective sperm entry, oocyte activation, pronuclear formation or fusion^34^, as well as some failure in mitotic division^35^. In this work, we profiled the transcriptome of the Poly-PN and PN arrest zygotes from two patients with RTFF, and found Poly-PN zygotes showed defects in Meiosis and RNA processing and PN arrest zygotes had defects in Cell cycle and DNA homologous recombination.

For meiosis, oocytes need to undergo meiotic DNA replication and homologous chromosomes segregation, and then arrest in metaphase of meiosis II awaiting fertilization^33^. After sperm penetration, oocyte resumes meiosis and segregates sister chromatids and completes the meiosis II. So the differentially expressed genes in Poly-PN might play critical roles in this biological process. For example, the subunit of the SCF E3 ubiquitin ligase (SKP1) has been reported to be important in the progression of recombination during oocyte meiosis^36^. The APC core subunit (CDC27) and the checkpoint protein (MAD2) play critical roles in segregating sister chromatids during oocyte meiosis^37^. Similarly, some other genes down regulated in Poly-PN zygotes, such as Protein Phosphatase (*PPP2R1B*), *YWHAZ* and *SPDYC* were also in associated with meiosis^38,39^.

Furthermore, during oocyte maturation, it also needs to accumulate sufficient maternal RNA to ensure oocyte maturation, fertilization and subsequently embryo development until the embryonic genome is activated^40^. So certain RNA processing genes identified in Poly-PN zygotes, such as those encoding splicing factor genes *THOC1*^41^, *SF3B1*^42^, *LOC645691*, exon junction complex core component related gene (*MAGOHB*)^43^, PHD finger-like domain-containing protein 5 A (*PHF5A*)^44^, some pre-mRNA processing factor 18 related gene (*PRPF18*)^45^ and RNA helicases related genes (*DDX5* and *BAT1*)^46,47^, are involved in regulating RNA secondary structure and pre-mRNA splicing, which might be responsible for RNA maturation during oocyte meiosis.

**Figure 5:**
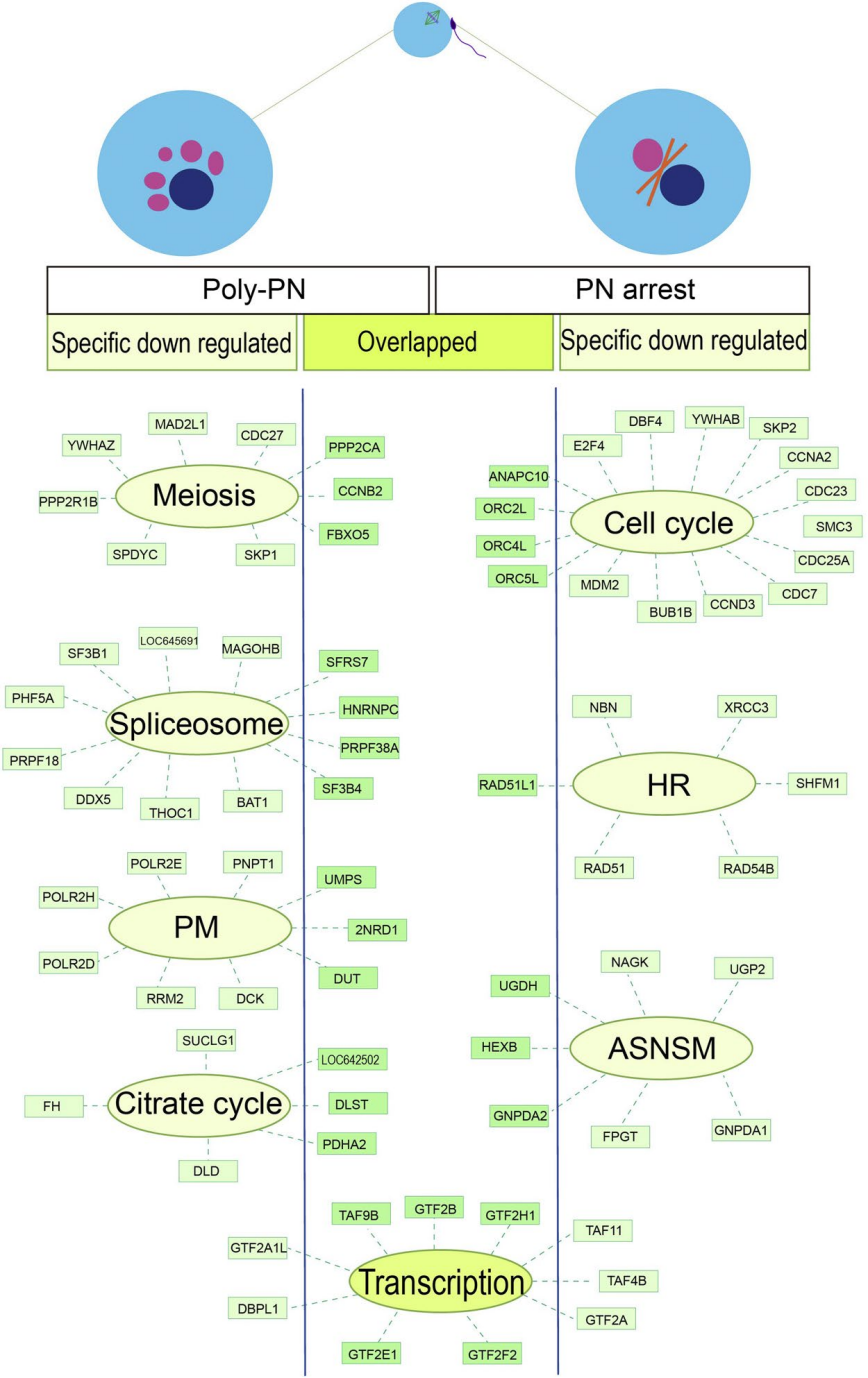
Module visualization of Poly-PN and PN arrest specifically down regulated SDEGs. Module visualization of Poly-PN and PN arrest specifically down regulated SDEGs. Down regulated SDEGs were shown Poly-PN specifically (left panel) PN arrest specifically (right panel) and genes down regulated overlap in both of this two groups (middle panel), PM, Pyrimidine metabolism; HR, Homologous recombination; ASNSM, Amino sugar or nucleotide sugar metabolism.

Upon fertilization, the zygotes undergoes chromatin remodeling, genomes reprogramming or DNA repairing, and the cell cycle machinery must be switched from meiotic to mitotic chromosome segregation^48^. Our results indicated that the PN arrest specific down regulated genes mainly related to these biological process. For example, the cyclin associated kinase (*CCNA2* and *CCND3*) were required for sister chromatid segregation^49^, and structural maintenance and segregation of chromosome proteins (*SMC3* and *BUB1B*) have been reported to be in associated with developmental potential of human pre-implantation zygotes^50^. Cell cycle related genes (*CDC7*, *CDC23* and *CDC25A*) and some other genes including *DBF4, YWHAB, SKP2 and MDM2* were also found to be significantly down regulated in PN arrest group. Moreover, some other genes specifically down-regulated in PN arrest zygotes, including check point proteins codon genes (*RAD54B* and *RAD51*)^51,52^, DNA repair related genes (*XRCC3*)^53^, chromosome integrity maintenance genes (*NBN*)^54,55^ and *SHFM1*, all of which were involved in key proteins for homologous recombination. In addition, we also found some genes down regulated overlap for both Poly-PN and PN arrest groups and these genes in different annotations were classified according to the expression specification and illustrated in a model (Fig. 5).

Taken together, our work found Poly-PN have some problems in oocyte meiosis and RNA processing, whereas PN arrest showed defects during mitosis cell cycle or homologous recombination during meiosis and this could provide new targets for therapeutic intervention by modulating these corresponding signaling pathways in the future. Remarkably, as the scarce of the RTFF patients, we could not collect enough oocyte samples for single cell RNA sequencing. So more clinical cases need to be collected and further verification need to be performed in the future.

## Methods

### Ethics statement

All procedures were approved by the Research Ethics Committee of Shanghai Jiao tong University School of Medicine and informed consent was obtained from participants at IVF center of the Ninth people’s hospital. We confirmed that all patients have written informed consent for the use of their zygotes for this research. Animals were maintained at 23 °C in a 12-h (7:00–19:00) light and 12-h (19:00–7:00) dark schedule, and all experimental procedures were performed in accordance with Institutional Animal Care and Use Committee guidelines of Shanghai Jiao Tong University School of Medicine.

### Patients, ovarian stimulation, oocyte retrieval, and the IVF/ICSI procedure

For all patients in our study, five types of ovarian stimulation protocols were used: (1) Human Menotrophins Gonadotrophin (hmG, Lizhu Pharmaceutical Trading Co.) co-treated with Medroxyprogesterone acetate (MPA, Shanghai Sine Pharmaceutical Ltd.) (hMG + MPA); (2) Human Menotrophins Gonadotrophin co-treated with Clomifene Citrate (CC, Medochemie Ltd.) (hMG + CC); (3) Human Menotrophins Gonadotrophin co-treated with Medroxyprogesterone acetate and Clomifene Citrate (hMG + MPA + CC); (4) Human Menotrophins Gonadotrophin co-treated with Medroxyprogesterone acetate and ethinyl estradiol (EE, Shanghai Sine Pharmaceutical Ltd.) (hMG + MPA + EE) and (5) Short protocol, in which patients were administered with GnRHa daily beginning on menstrual cycle day 2 and with hMG daily beginning on menstrual cycle day 3. Follicle growth was monitored by ultrasound examination. Serum FSH, LH, E_2_, and progesterone concentrations were measured serially using the chemiluminescence (Abbott Biologicals B.V.) method on the same days as the ultrasound exams. Human Chorionic Gonadotrophin (hCG, Lizhu Pharmaceutical Trading Co.) at a 1000–5000 IU dose was administered when the dominant follicles reached 18 mm in diameter. Cumulus oocyte complexes were recovered transvaginally with ultrasound guidance 34–36 hours post hCG. After retrieval, oocytes were maintained in human tubal fluid (HTF; Irvine Scientific) medium plus 10% synthetic serum substitute (SSS; Irvine Scientific) for about 2 hours before *In vitro* fertilization (IVF)/Intracytoplasmic sperm injection (ICSI).

For ICSI treatment, the cumulus oophorus were removed mechanically from oocytes with denuding pipettes in solution with 80IU hyaluronidase (Sigma) followed by injection. For IVF treatment, cumulus oocyte complexes were inseminated with about 0.3–0.5 × 10^6^/ml motile spermatozoa in HTF medium and the cumulus oophorus were removed 18 hours later. Fertilized eggs from both IVF and ICSI groups were cultured in 20 μl continuous single culture medium (CSC: Irvine Scientic: USA) individually under oil and incubated at 37 °C humidified atmosphere under 5% CO_2_, 5% O_2_, and 90% N_2_ for pre-implantation culture. As a policy of our center, fertilization was assessed by the presence of two pronuclei 16–18 hours post insemination, followed by confirming the embryonic development 66–68 hours post insemination. The zygotes with more than three tiny pronuclei following the ICSI procedure were recognized as Poly-PN zygotes. The zygotes with normal pronuclei but failed to fuse until 66–68 hours post fertilization were name as PN-arrest zygotes. Tri-PN zygotes from four different IVF patients were used as controls. All samples above were collected and vitrified using Cryotip method and then stored in liquid nitrogen until subsequent experimental treatment.

### Preparation and quality control of single-cell cDNAs

The method for RNA extraction was carried out as described previously^56^. Briefly, after thawing, each zygote was washed twice and transferred into lysate buffer. Then the reverse transcription reaction was performed directly on whole cell lysate using SuperScript II reverse transcriptase (Life Technologies). We performed 15 cycles of PCR to amplify cDNA and the PCR product was purified by using AMPure XP beads (Beckman Coulter). Agilent high-sensitivity DNA chip kit on a BioAnalyzer (Agilent Technologies) was used for checking the quality of cDNAs according the size distribution to ensure cDNAs contained few short fragments (<500 bp) and showed peak sizes between 1.5 kb–2 kb.

### RNA-Seq library construction and sequencing

According to the manual of TruePrep DNA Library Prep Kit V2 for Illumina (Vazyme Biotech), the quality of RNA-Seq sequencing library was checked by using Agilent high-sensitivity DNA chip. The libraries showing the peak around 300 bp was chosen for high-throughput sequencing on the Illumina HiSeq 2500 platform using the dual index sequencing strategy with single-end reads length of 50 bp.

### Bioinformatics process for sequencing data

Individual sample from different zygotes has its own unique barcode sequence and could be separated from clean data. We used Tophat v2.0.9^57^ to assemble the reads into NCBI build 37 hg19 genome and used Cufflinks v2.1.1^58^ to calculate gene expression level. Clustering was used to process hierarchical clustering using Euclidean distance metric in the R packages^59^. Gene expression levels were measured by using fragment per kilobase of exon per million mapped reads (FPKM). To rule out technical errors and increase the power to detect biological function, all reference genes with average FPKM > 0.5 in any of three groups and the criterion of *P* < 0.001 or *P* < 0.01 together with FC (fold change) >2 or <0.5 were used to identify differentially expressed genes for subsequent biological analysis using ArrayTrackTM software (FDA’s own bioinformatics and genomics tool, http://www.fda.gov/ScienceResearch/BioinformaticsTools/Arraytrack/default.htm).

### KEGG pathway analysis

Database for Annotation Visualization and Integrated Discovery (DAVID V6.7; https://david.ncifcrf.gov/) was used to perform KEGG pathway analysis^60,61^.

### ShRNA design and ***in vitro*** transcription

For short hairpin RNA (shRNA) design, we selected an siRNA-target sequence on the NCBI RNAi database for each targeted genes, and the forward and reverse primers for each gene (SKP1 F: ATAGGGGGCT GCAAACTACT TAGACATTTC AAGAGA ATGT CTAAGTAGTT TGCAGCCTTT TTTG; SKP1 R: GATCCAAAAA AGGCTGCAAA CTACTTAGAC ATTCTCTTGA AATGTCTAAG TAGTTTGCAG CCCC; PPP2CA F: ATAGGGTGGA ACTTGACGAC ACTCTTATTC AAGAGATAAG AGTGTCGTCA AGTTCCATTT TTTGPPP2CA R: GATCCAAAAA ATGGAACTTG ACGACACTCT TATCTCTTGA ATAAGAGTGT CGTCAAGTTC CACC) were annealed and cloned into a T7 promoter containing vector pcDNA3.1(+) using Bsa1 and BamH1 restriction enzyme site, shRNA was transcribed *in vitro* from linearized pcDNA3.1-shRNA plasmid using MEGA short script T7 kit (Life Technology) and purified using MEGA clear kit (Life Technology) and mixed in RNase-free water at the concentration of 50 ng/μl for subsequent use.

### Oocyte microinjection, parthenogenetic activation and development assessment

Female mice aged 6–8 weeks were induced to superovulate by i.p. injection of 10 IU of pregnant mare’s gonadotrophin (PMSG) (Ningbo Hormone Products Co.). Cumulus oocyte complexes (COCs) were collected at 46 h post PMSG. For COCs retrieval, the ovaries were removed immediately and put into 4 ml HTF medium plus 10% SSS (Irvine Scientific) and 0.2 mM IBMX (Sigma). The COCs were released into this medium by puncturing ovaries with a 27 g needle. The cumulus cells were released mechanically using mouth pipette and only those with normal morphologies were used for RNA injection.

All injected oocytes were cultured for maturation in a CO_2_ incubator for 16 hours for maturation before activation. The activation medium used was KSOM (Millipore) supplemented with 10 mM SrCl_2_. After being washed twice in activation medium, oocytes were incubated first in activation medium for 2.5 hours and then in activation medium without SrCl_2_ for 3.5 hours at 37 °C in a humidified atmosphere with 5% CO_2_, 5% O_2_, and 90% N_2_. Both the activation medium and KSOM for subsequent short culture of oocytes were supplemented with 5 μg/mL cytochalasin B. Six hours after the onset of activation, the fertilization rate was assessed by count pronuclear formation.

### Statistical analysis

Serum hormone data were analyzed by GraphPad Prism software using two-way repeated measures ANOVA. Bonferroni post tests were used for pairwise comparisons. **P < 0.01; ***P < 0.001. Data for relative expression levels in Poly-PN and PN arrest zygotes were separately compared with control were analyzed using a two-tailed, unpaired Student’s t test. *P* < 0.001 indicated as significantly different. The maturation and fertilization rate of the oocytes between gene knock down and control group were analyzed by using Chi-Squared Test.

## Electronic supplementary material

Supplementary information

Dataset 1

Dataset 2

Dataset 3

Dataset 4

Dataset 5

Dataset 6
