# Supplementary material for: Transcriptome profiling of human oocytes experiencing recurrent total fertilization failure
Source: Sci Rep. 2018 Dec 17;8:17890. doi: 10.1038/s41598-018-36275-6 (PMC6297154; doi:10.1038/s41598-018-36275-6)
Supplement: Supplementary file 1 — Supplementary information [file 41598_2018_36275_MOESM1_ESM.pdf]

Transcriptome profiling of human oocytes experiencing recurrent total fertilization failure

Lun Suo[1], Yu xiao Zhou[2], Li ling Jia[2], Hai bo Wu[1], Jin Zheng[2], Qi feng Lyu[1], Li hua Sun[1], Han Sun[3] & Yan ping Kuang[1]

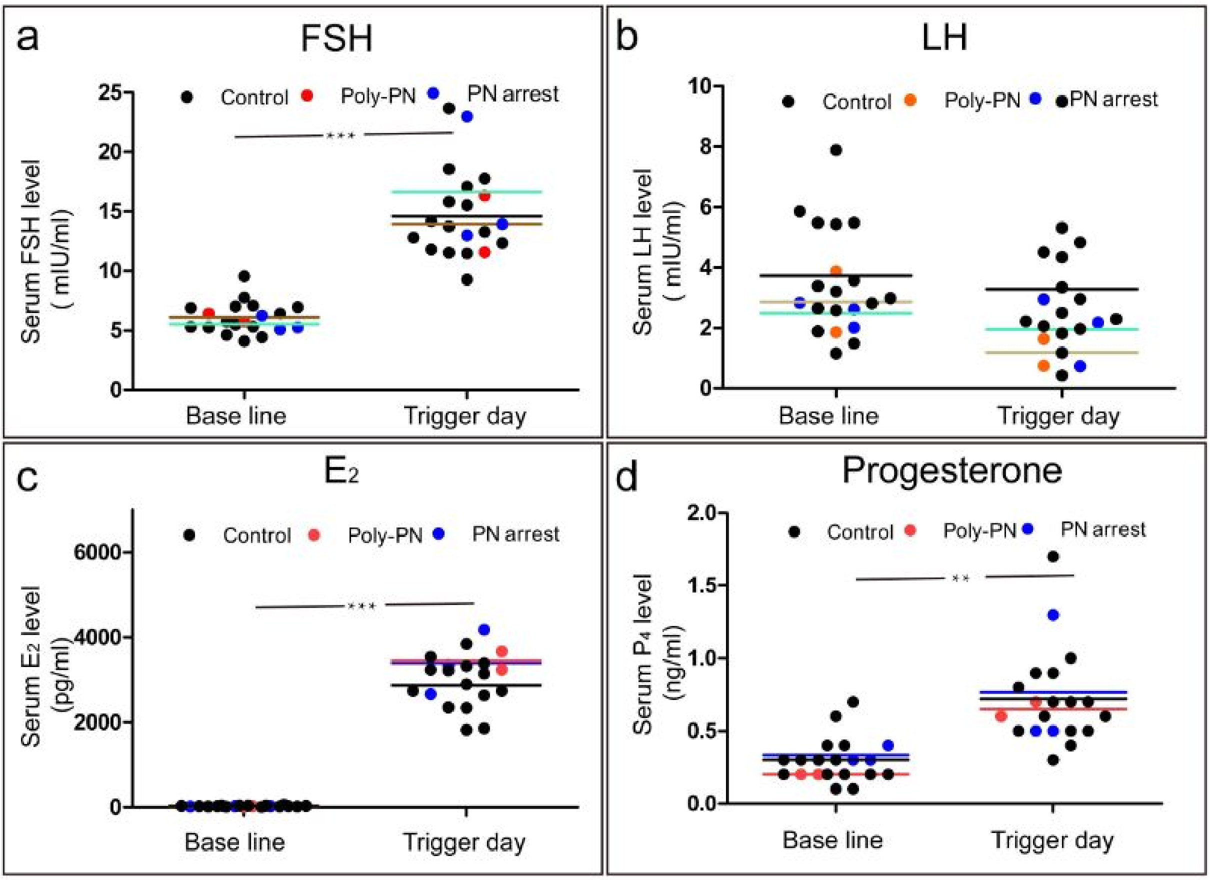

Supplementary figure 1. Serum hormone levels separately at base line and trigger day during ovarian stimulation cycles. (a-d) Serum hormone levels were significantly changed in same group at two time point but without significant difference between groups in FSH ($F_{times}(1,17) = 55.81$, $P < 0.0001$; F_{groups} $(2,17) = 0.18$, $P = 0.83$)(a)**,** E_2 ($F_{times}(1,17) = 286.36$, $P < 0.0001$; $F_{groups}(2,17) = 1.49$, $P = 0.25$)(c) and Progesterone ($F_{times}(1,17) = 9.80$, $P < 0.01$; $F_{groups}(2,17) = 0.43$, $P = 0.66$)(d), as well as any difference was not shown in either time points and groups for LH ($F_{times}(1,17) = 2.50$, $P = 0.13$; $F_{groups}(2,17) = 1.22$, $P = 0.32$)(b).
